# The utility of low-iodine diet in preparation for thyroid cancer therapy with radioactive iodine—A cohort study

**DOI:** 10.3389/fphar.2022.791710

**Published:** 2022-09-30

**Authors:** Hongxiu Luo, Andrew Tobey, Sungyoung Auh, Craig Cochran, Noha Behairy, Maria Merino, Marina Zemskova, Joanna Klubo-Gwiezdzinska

**Affiliations:** ^1^ National Institute of Diabetes and Digestive and Kidney Diseases, National Institutes of Health, Bethesda, MD, United States; ^2^ Saint Peter’s University Hospital, New Brunswick, NJ, United States; ^3^ National Cancer Institute, National Institutes of Health, Bethesda, MD, United States

**Keywords:** thyroid cancer, urinary iodine excretion, radioactive iodine, progression, low-iodine diet

## Abstract

**Objective:** A low-iodine diet (LID) of <50μ iodine/day is recommended as preparation for radioactive iodine (RAI) therapy in patients with differentiated thyroid cancer (DTC). The 24-h urinary iodine excretion (UIE) is utilized to evaluate the iodine-depleted status. The aim of this study was to test the association between UIE and progression-free survival (PFS).

**Patients and methods:** In total, 70 patients with intermediate- or high-risk DTC, post-total thyroidectomy, adhered to 2 weeks of LID and had UIE measured before RAI therapy. A Cox regression model was performed to study the contribution of UIE to PFS.

**Results:** The study group consisted of 68% (48/70) of women, aged 41.5 [IQR 31.0, 54.0] years, with tumor size 2.8 [IQR 1.8–4.5] cm, and presence of distant metastases in 22.9% (16/70) of patients. Patients were treated with 1–5 RAI dosages with the median cumulative activity of 150 [IQR 102–314] mCi (5.5 [IQR 3.8–11.6] GBq). During the follow-up of 3.7 [IQR 1.5–6.5] years, 21.4% (15/70) of patients had disease progression. The risk of progression was significantly higher in patients with UIE ≥200 µg/day at the time of RAI administration than in those with UIE <200 µg/day (HR 3.35, 95% CI 1.09–10.34, and *p* = 0.02). However, the multivariate Cox proportional hazards regression analysis adjusted for age, tumor size, and presence of distant metastases suggested that only distant metastases were independently significantly associated with the risk of progression (HR 5.80 (1.17–28.67), *p* = 0.03).

**Conclusions:** Although UIE ≥200 µg/day might be associated with worse PFS in RAI-treated DTC patients, the presence of distant metastases is a strong independent predictor of progression. Less stringent LID might be sufficient to achieve a UIE of <200 µg/day.

## Introduction

Thyroid cancer is an endocrine malignancy characterized by a multifactorial etiology consisting of contribution from somatic drivers such as *BRAF*-like and *RAS*-like molecular signatures, combined with a contribution from environmental factors such as the increased prevalence of obesity and presence of chemical thyroid disruptors in the environment that modify the genetic background of this malignancy (Kitahara et al., 2019; Marotta et al., 2020) (Lim et al., 2017). The therapeutic approach to thyroid cancer is based on a risk stratification system—the likelihood of death from thyroid cancer and the probability of persistent/recurrent disease. When low-risk patients with thyroid cancer, with the disease confined to the thyroid, are characterized by an excellent response to surgical treatment, not warranting any adjuvant therapies, patients with locally advanced disease or distant metastases often require adjuvant treatment (Haugen et al., 2016). The current American Thyroid Association guidelines recommend that the therapy of locally advanced or metastatic differentiated thyroid cancer (DTC) should consist of total thyroidectomy, followed by radioactive iodine (RAI) treatment (Haugen et al., 2016). It has been shown that RAI therapy may prolong overall survival and disease-free survival and reduce long-term cancer recurrence in high-risk patients with DTC.

In order to optimally prepare for RAI therapy, an elevated concentration of thyroid-stimulating hormone (TSH or thyrotropin) is required as it stimulates the expression of the sodium–iodine symporter in cancer cells, leading to a higher RAI uptake. There are two possible methods for TSH stimulation. The first one is stimulation with a recombinant human TSH (rhTSH) injection, and the second consists of thyroid hormone withdrawal (THW) to provoke endogenous TSH elevation (Haugen et al., 2016). In addition to TSH stimulation, a low-iodine diet (LID) implemented prior to RAI therapy can be used to enhance RAI uptake. However, the stringency and duration of the iodine restriction vary between different sets of guidelines. Low dietary iodine of less than 50 µg/day for 1–2 weeks is currently recommended by the American Thyroid Association for patients undergoing RAI remnant ablation (Haugen et al., 2016). The stringency of an LID is not specified by the British Thyroid Association which recommends an LID for 1–2 weeks (Mitchell et al., 2016), similar to the European Thyroid Cancer Taskforce, which recommends an LID for 3 weeks without specifying its stringency (Pacini et al., 2006; Sawka et al., 2010). Although the majority of studies on the efficacy of the LID limit daily iodine intake to less than 50 µg/day (Morris et al., 2001; Pluijmen et al., 2003; Haugen et al., 2016; Li et al., 2016), there is no clear consensus amongst the guidelines. Moreover, there are limited data examining the impact of the LID used for preparation for RAI therapy on the long-term progression of DTC or mortality rates (Sawka et al., 2010). Therefore, the goal of this study was to investigate whether urinary iodine excretion (UIE) is associated with progression-free survival (PFS) in patients with intermediate- and high-risk DTC, who underwent total thyroidectomy with or without central/lateral lymph node dissection and RAI therapy.

## Materials and methods

### Study design and study population

A retrospective cohort study including patients with DTC was performed at the National Institute of Health (NIH) after obtaining approval from the Institutional Review Board (clinicaltrials.gov ID NCT00001160). All patients had to be fulfilling the following inclusion criteria: 1) confirmed diagnosis of DTC based on the pathology of the tumor tissue obtained after total or near-total thyroidectomy; (patients with more aggressive histology of thyroid tumors of epithelial origin were included if they maintained the expression of thyroid-specific genes such as thyroglobulin (Tg) and RAI uptake on diagnostic and/or post-therapy scans); 2) intermediate- or high-risk patients based on the ATA risk stratification; 3) treatment with RAI under either THW or rhTSH preparation for thyrotropin stimulation with a diagnostic scan performed before therapy and a post-therapy scan performed 5–7 days posttreatment; 4) at least one complete follow-up examination including suppressed and/or stimulated Tg levels, ultrasound of the neck, I^123^ or I^131^ whole body scan, chest CT, or other imaging techniques, as appropriately performed every 6–12 months after treatment. All patients were recommended to adhere to an LID for at least 2 weeks prior to the RAI treatment with the goal of urine iodine of <150 µg/day. In-person counseling about the diet and written instructions and recipes including low-iodine products were given to the patients. Patients were followed with a median follow-up time of 3.7 years.

### 24-h urine collection and measurements of urinary iodine excretion

All participants were asked to collect 24-h urine before each RAI dosage as this is a gold standard to assess the iodine-repleted/depleted status (Baloch et al., 2003). Details of the collection method have been reported elsewhere (Katagiri et al., 2016). Briefly, participants set the starting time of collection and then discarded the first urine at the starting time. All subsequent specimens were collected until the same time as the starting time on the next day. The urinary iodine content of the collected urine was measured at Mayo Labs and analyzed by inductively coupled plasma mass spectrometry (ICP-MS) in the standard mode using tellurium (Te) as an internal standard and an aqueous acidic calibration.

### Covariates

Demographic information on age at diagnosis, gender, and tumor characteristics including the histology subtype, tumor size, and presence of loco-regional or distant metastases, as well as the method of TSH stimulation before RAI, RAI uptake in the posttreatment scan, and cumulative RAI dose, was extracted from medical records.

### Outcomes

The primary outcome was progression-free survival (PFS). PFS was defined as time from the initial diagnosis to the first evidence of structural disease progression. Per RECIST 1.1 criteria, the structural disease progression was defined as at least a 20% increase in the sum of three diameters of target lesions, taking as a reference the smallest sum in the study and an absolute increase of at least 5 mm or appearance of one or more new lesions (Eisenhauer et al., 2009; Goebel et al., 2017).

The secondary outcome was the best overall response to treatment. According to 2015 ATA guidelines, we categorized the overall response as 1) excellent response (ER)—negative imaging and suppressed Tg < 0.2 ng/ml or stimulated Tg < 1 ng/ml, 2) biochemical incomplete response (BIR)—negative imaging and suppressed Tg > 1 ng/ml or stimulated Tg > 10 ng/ml or rising anti-Tg Ab levels, 3) structural incomplete response (SIR)—structural or functional evidence of disease with any Tg level+/F02DTg Ab, and 4) indeterminate response (IR)—nonspecific findings on imaging studies, faint uptake in thyroid bed on RAI scanning, non-stimulated detectable Tg but less than 1 ng/ml, and stimulated detectable Tg but less than 10 ng/ml or Tg antibodies stable or declining in the absence of structural or functional disease (Haugen et al., 2016).

### Statistical analysis

We summarized the baseline demographics and clinical characteristics using either median with the 25%–75% interquartile range (IQR) or proportions as appropriate. The study cohort was stratified into groups based on 24-h urine iodine excretion with the threshold established at 50, 100, 150, 200, and 250 µg/24h. To compare the baseline characteristics between the groups, Kruskal–Wallis and chi-squared tests were used for continuous variables and categorical variables, respectively. In addition, Kaplan–Meier survival analyses were performed to compare time to progression (progression-free survival—PFS) between the groups. We also used the Cox proportional hazards regression model to study the contribution of age, tumor size, presence of distant metastases, and cumulative RAI dose with a stepwise variable selection for PFS. Estimated hazard ratios (HRs) with corresponding 95% confidence intervals (CIs) were reported using the univariate and multivariate Cox proportional hazards models. All analyses were two-tailed tests based on *α* = 0.05 and were conducted by SAS version 9.4 (SAS Institute, Cary, NC).

## Results

The study cohort consisted of 70 intermediate- and high-risk DTC patients (48 women, 22 men) characterized by a median age at a diagnosis of 41.5 [IQR 31.0, 54.0] years and tumor size 2.8 [IQR 1.8, 4.5] cm with the presence of distant metastases in 22.9% (16/70) of patients. Among patients with distant metastases, 4 out of 16 presented with pulmonary micro-metastases, 7 of 16 with pulmonary micro- and macro-metastases, 1 of 16 patients with pulmonary macro-metastases, and 4 of 16 patients with lung and bone metastases. The patients were treated with one to five RAI dosages with the median cumulative activity of 150 [IQR 102–314] mCi (5.5 GBq [IQR 3.8–11.6])—16 patients prepared with THW and 54 with rhTSH. All patients received the first RAI dose, 23 patients received two RAI dosages, 15 patients received three RAI dosages, 3 patients were treated with four RAI dosages, and 1 patient received five RAI treatments over the course of the follow-up. The first RAI dose ranged from 30 mCi (1.1 GBq) (for intermediate-risk patients) to 300 mCi (11.1 GBq) (dosimetry-based dose for high-risk patients), while the subsequent RAI dosages ranged from 150 mCi (5.5 GBq) to 315 mCi (11.6 GBq). Post-first RAI therapy, ^131^I whole-body scans (^131^I-WBS) revealed RAI-avid disease and/or thyroid remnant uptake in all 70/70 patients after the first RAI dosage; post-second RAI dose, the ^131^I-WBS revealed RAI-avid disease in 20/23 patients; post-third RAI therapy, the ^131^I-WBS revealed RAI-avid disease in 14/15 patients; post-fourth RAI therapy, the ^131^I-WBS revealed RAI-avid disease in 3/3 patients; and one patient treated with five RAI dosages had a positive post-therapy ^131^I-WBS. Pretreatment urine iodine in patients presenting with decreased RAI uptake over the course of the follow-up was not significantly different compared with the patients with preserved over time RAI-avid disease [median 41 (25–75% IQR 27–90.5) vs. 60 (25–75% IQR 32.5–108.5), *p* = 0.55]. Representative images of post-treatment scans of intermediate- and high-risk patients who had either an excellent response to therapy or presented with disease progression have been disclosed in Supplementary Figure S2. Although the patients prepared for RAI with THW tended to have lower 24-h UIE than the patients undergoing rhTSH injections, the difference was not statistically significant [46.5 (29.8–85) vs. 88 (45–168) µg/day, *p* = 0.2].

Over the course of the follow-up, 21.4% (15/70) of patients had structural disease progression. The evidence of structural disease progression, corresponding tumor marker (Tg and anti-Tg antibodies) levels, and the intervention made once progression has been established are summarized in Supplementary Table S1.

There was no difference in age at diagnosis, gender breakdown, or at the presence of a gross extrathyroidal extension between patients with and without structural disease progression (Table 1). Patients with progression had a larger tumor size (*p* = 0.046), higher prevalence of distant metastases (*p* < 0.001), and higher cumulative RAI dose (*p* < 0.001) compared to patients without progression (Table 1). No significant difference in the duration of the follow-up was observed between the two study groups (Table 1).

**TABLE 1:** Baseline characteristics of the study groups.

Baseline characteristic	Overall (n = 70)	Patients with progression		*p*-value
		Yes (n = 15)	No (n = 55)	
**Age at diagnosis (median, IQR, Yr)**	41.5 (31.0–54.0)	49.0 (35.0–60.0)	40.0 (30.0–49.0)	0.18
**Gender (no. of patients, %)**				0.86
Female	48 (68.6)	10 (66.7)	38 (69.1)	
Male	22 (31.4)	5 (33.3)	17 (30.9)	
**Histology (no. of patients, %)**				0.17
Follicular thyroid cancer	3 (4.3)	1 (6.7)	2 (3.6)	
Hurtle cell thyroid cancer	5 (7.1)	1 (6.7)	4 (7.3)	
Poorly differentiated thyroid cancer*	2 (2.9)	2 (13.3)	0 (0.0)	
Papillary thyroid cancer (tall-cell variant)	19 (27.1)	5 (33.3)	14 (25.5)	
Papillary thyroid cancer (follicular variant)	13 (18.6)	2 (13.3)	11 (20.0)	
Papillary thyroid cancer (classic)	28 (40.0)	4 (26.7)	24 (43.6)	
**Tumor size (median, IQR, cm)**	2.8 (1.8–4.5)	4.7 (2.5–6.0)	2.7 (1.8–4.2)	0.046
**Cumulative RAI dose (median, IQR, mCi) [GBq]**	150 (102–314)	524 (359–590)	150 (100–200)	<0.001
	[5.5 (3.8–11.6)]	[19.4 (13.3–21.8)]	[5.5 (3.7–7.4)}	
**Urinary iodine excretion (UIE) (median, IQR, µg/day)**	71 (34–140)	140 (58–252)	52 (32–120)	0.006
**Distant metastasis**				<0.001
Yes	16 (22.9)	9 (60.0)	7 (12.7)	
No	54 (77.1)	6 (40.0)	48 (87.3)	
**Duration of the follow-up (median, IQR, Yr)**	3.7 (1.5–6.5)	4.4 (2.0–9.5)	3.6 (1.4–5.9)	0.14

* Patients with a component of poorly differentiated histology maintained the expression of thyroid-specific genes such as thyroglobulin (Tg) and RAI, and uptake on diagnostic and/or post-therapy scans, as documented in Supplementary Figure S1.

**FIGURE 1:**
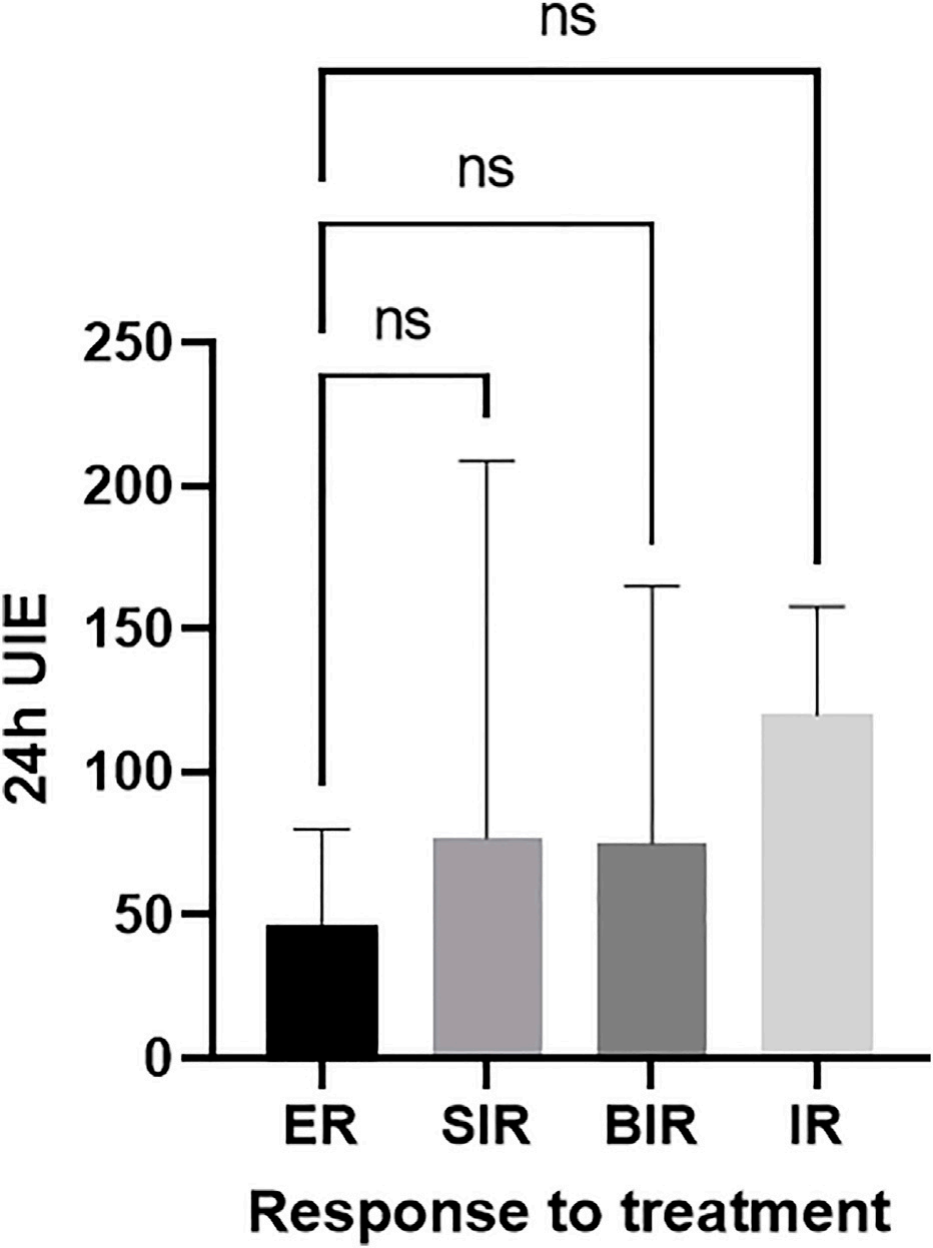
No difference between the 24-h UIE before RAI and best overall response to treatment (ER-reference group). ER, excellent response; BIR, biochemical incomplete response; SIR, structural incomplete response; IR, indeterminate response.

During the follow-up of 3.7 [IQR 1.5–6.5] years, 35/70 (50%) of patients had an ER, 21/70 (30%) patients had a SIR, 3/70 (4.3%) had a BIR, 11/70 (15.7%) had an IR, and 21.4% (15/70) of patients had disease progression. However, no significant difference in median 24-h UIE was observed in patients with ER (47 [23–108] µg/day), SIR (70 [30–210] µg/day), BIR (75 [29–165] µg/day), and IR (120 [45–140] µg/day), *p* = 0.51 (Figure 1).

**FIGURE 2:**
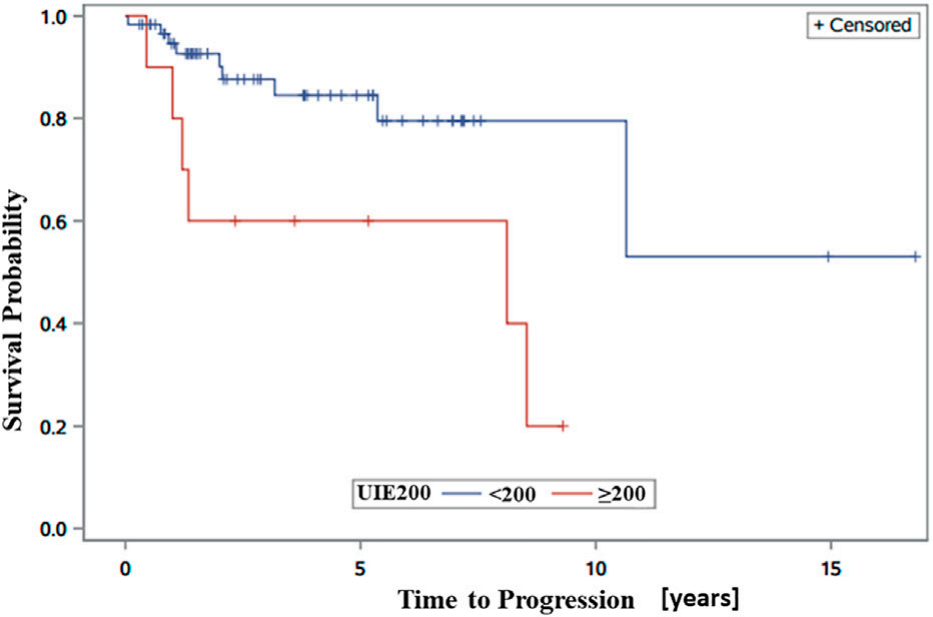
Kaplan–Meier progression-free survival (PFS) curves showing significantly better PFS in patients with UIE <200 than in those with UIE ≥200 µg/day.

We observed that patients with progression had a relatively higher average 24-h UIE before RAI than patients without progression (Table 1). Therefore, we next analyzed the association between different cutoffs of UIE and PFS. When we set the cutoff of UIE at 50 µg/day, 100 µg/day, or 150 µg/day, no significant association between UIE and PFS was observed in the univariate analysis (Table 2). However, patients with UIE ≥200 µg/day were characterized by a higher risk of progression than those with UIE <200 µg/day (HR 3.55, CI 1.18–10.61, and *p* = 0.02) (Table 2; Figure 2).

**TABLE 2:** Relative hazard ratios (HRs) and 95% confidence interval (CI) of progression for urinary iodine excretion (UIE) using the univariate Cox proportional regression analysis.

Categorized UIE (µg/day)	Hazard ratio (95% CI)	*p*-value
UIE50 (cutoff 50 µg/day)		
≥ 50 vs. < 50	3.22 (0.70–14.73)	0.13
UIE100 (cutoff 100 µg/day)		
≥ 100 vs. < 100	1.82 (0.64–5.19)	0.26
UIE150 (cutoff 150 µg/day)		
≥ 150 vs. < 150	2.28 (0.79–6.57)	0.13
UIE200 (cutoff 200 µg/day)		
≥ 200 vs. < 200	3.55 (1.18–10.61)	0.02^*^
UIE250 (cutoff 250 µg/day)		
≥ 250 vs. < 250	3.79 (1.17–12.32)	0.03^*^

* *p* < 0.05.

A multivariate Cox proportional hazards model including urine iodine, age, tumor size, cumulative RAI dose, and presence of distant metastases revealed that only distant metastases were significantly associated with the risk of progression (HR 5.64 (1.07–25.41), *p* = 0.03) (Table 3). Therefore, we next performed a subgroup analysis in patients who presented with distant metastases and subjects without distant metastases (Table 4). A significant association between UIE ≥200 µg/24 h and PFS was found in patients without distant metastasis (HR 5.96, CI 1.00–35.74, and *p* = 0.05; adjusted HR 6.83, CI 1.13–41.19, and *p* = 0.01) but not in patients with distant metastasis (Table 5). These data confirmed that UIE ≥200 µg/24 h is a significant factor associated with PFS, but the presence of distant metastases has a stronger association with PFS than 24-h UIE. Since only one patient died during the follow-up, we did not further evaluate the overall survival.

**TABLE 3:** Relative hazard ratios (HRs) and 95% confidence interval (CI) of progression for urinary iodine excretion (UIE) using the multivariate Cox proportional regression analysis.

Categorized UIE (µg/day)	Hazard ratio (95% CI)	*p-*value
UIE200 (cutoff 200 µg/day)		
Age	0.96 (0.93–1.04)	0.45
Tumor size	1.08 (0.71–1.68)	0.56
Cumulative RAI dose	1.03 (0.64–2.12)	0.74
Distant metastasis	5.64 (1.07–25.41)	0.03^*^
≥ 200 vs. < 200	1.62 (0.30–7.54)	0.57

* *p* < 0.05.

**TABLE 4:** Patient characteristics stratified by the presence of distant metastasis.

Baseline characteristic	Distant metastasis		*p*-value
	Yes (*n* = 16)	No (*n* = 54)	
**Age at diagnosis (median, IQR, Yr)**	45.5 (35.0–56.5)	41.0 (29.0–49.0)	0.41
**Gender (no. of patients, %)**			0.21
Female	13 (81.3)	35 (64.8)	
Male	3 (18.8)	19 (35.2)	
**Histology (no. of patients, %)**			0.17
Follicular thyroid cancer	1 (6.3)	2 (3.7)	
Hurtle cell thyroid cancer	1 (6.3)	4 (7.4)	
Poorly differentiated thyroid cancer	2 (12.5)	0 (0.0)	
Papillary thyroid cancer (tall-cell variant)	4 (25.0)	15 (27.8)	
Papillary thyroid cancer (follicular variant)	4 (25.0)	9 (16.7)	
Papillary thyroid cancer (classic)	4 (25.0)	24 (43.4)	
**Tumor size (median, IQR, cm)**	5.0 (3.2–6.0)	2.5 (1.5–4.0)	<0.001
**Cumulative RAI dose (median, IQR, mCi) [GBq]**	425 (203–690)	150 (100–200)	<0.001
	[15.7 (7.5–25.5)]	[5.5 (3.7–7.4)]	
**Urinary iodine excretion (UIE) (median, IQR,** F06D**g/24h)**	109 (41–210)	62 (34–125)	0.11
**Categorized UIE (ug/24h)**			0.03
< 200	11 (68.8)	49 (90.7)	
≥ 200	5 (31.2)	5 (9.3)	
**Duration of the follow-up (median, IQR) years**	2.8 (1.1–4.9)	3.97 (1.5–7.0)	0.24

**TABLE 5:** Relative hazard ratios (HRs) and 95% confidence interval (CI) of progression for urinary iodine excretion (UIE) stratified by distant metastasis using the Cox proportional regression analysis.

Categorized UIE (µg/day)	Patients with distant metastasis		Patients without distant metastasis	
	Unadjusted HR (95% CI)	Adjusted HR (95% CI)^a^	Unadjusted HR (95% CI)	Adjusted HR (95% CI)^a^
UIE200 (cutoff 200 µg/day)				
**≥ 200 vs. < 200**	0.63 (0.12–3.28)	0.53 (0.10–2.76)	5.96 (1.00–35.74)^*^	6.83 (1.13–41.19)*

a Adjusted for age, tumor size, and cumulative RAI dose.

* *p* ≤ 0.05.

## Discussion

Our study showed that 24-h UIE ≥ 200 µg/day was associated with a higher risk of progression and shorter time to progression in patients with intermediate-to-high-risk DTC. However, the strong association between the presence of distant metastases at diagnosis and PFS may override the effect of UIE on PFS. To the best of our knowledge, this is the first study to evaluate the impact of UIE on PFS of DTC.

This observation has a physiologic rationale as there is evidence that the LID is associated with an enhanced uptake of RAI in thyroid remnant and metastatic lesions. Over four decades ago, Goslings (1975) found that the tumor uptake of RAI increased about 1.8 times and the effective half-life of RAI increased about 1.2 times after 4 days of LID in patients with metastatic follicular thyroid cancer. Similarly, Maruca et al. (1984) reported that after a 5-day LID, the tumor radiation dose significantly increased in two patients with metastatic thyroid cancer by 108% and 48%, respectively. Moreover, based on data provided by Pluijmen et al. (2003), the 24-h neck uptake of RAI was significantly higher in patients who were subjected to a LID for 4 days. Interestingly, Marotta et al. (2016) have recently discussed about the genetic and epigenetic alterations related to DTC that may impact iodine avidity and modulate the impact of the LID to enhance RAI uptake. However, it is worthwhile to recognize that aforementioned RAI kinetics, as a function of exposure to the LID, are based on data from a very limited sample size, disabling rigorous statistical comparisons. Thus, these results need to be interpreted with caution.

The next question is whether the reported enhanced uptake after an LID translates to a higher ablation success rate. There are significant discrepancies in the results of several studies examining the impact of the LID on the successful RAI ablation rate. Morris et al. (2001) reported no significant difference in ablation rates between 44 patients on a 2-week LID (68.2%) and 50 patients on a regular diet (62.0%). Similarly, Tala Jury et al. (2010) showed no significant relationship between 24-h UIE and the remnant ablation success rate, although the study was conducted in patients who were not specifically advised to adhere to an LID. Concordantly, Ito et al. (2018) revealed that in patients with intermediate-risk DTC who followed the current Japanese method of a 2-week LID before RAI, UIE was not a predictive factor for successful RAI ablation as documented at 6–8 months post-RAI treatment. In contrast, Pluijmen et al. (2003) conducted a study including 120 DTC patients without distant metastases who underwent RAI ablation after THW and found that a significantly higher proportion of patients (65%) who received a 4-day LID achieved successful ablation at 6 months than 48% of historical controls. Also, Sohn et al. (2013) retrospectively examined RAI ablation rates in association with urinary iodine/creatinine ratio (UICR, in µg/gCr) levels in 295 patients with papillary thyroid cancer who underwent THW and a 1–2-week LID post-surgery. They found that the ablation rate in those with UICR exceeding 250 µg/gCr at the time of RAI administration was significantly lower than that with UICR of less than 250 µg/gCr, while no significant differences in ablation rates were observed among subgroups with UICR <50, UICR 50–100, and UICR 100–250 µg/gCr (Sohn et al., 2013). The inconsistencies in the aforementioned studies may reflect heterogeneous baseline characteristics of included patients—with different dietary habits, variable UIE before a LID, different extents of initial surgery, variable age, presence of loco-regional or distant metastases, and different methods of TSH stimulation. The iodine-depleted status is associated with RAI uptake, which subsequently translates to treatment efficacy. A recent study utilizing I-124 PET/CT dosimetry revealed that the RAI uptake in metastatic lesions correlates with the RAI therapy success rate, with the dose-response threshold of 75Gy of RAI-absorbed dose per lesion associated with a complete or partial response (Plyku et al., 2022). It will be interesting to evaluate the effects of different UIE thresholds on the RAI uptake in metastatic foci utilizing I-124 PET/CT dosimetry. Our ongoing clinical trial will help address this question (clinicaltrials.gov ID NCT03841617). In addition to tumoricidal effects from beta radiation, iodine may also exert antiangiogenic effects on thyroid cancer (Daniell and Nucera, 2016).

Nutritional iodine sufficiency, reflecting consumption of 150 µg of iodine per day, based on the urinary iodine concentration (UIC) is defined as follows: optimal iodine nutrition—UIC of 100–199 μg/L, mild iodine deficiency—UIC of 50–99 μg/L, moderate deficiency—UIC of 20–49 μg/L, and severe deficiency—UIC less than 20 μg/L (Caldwell et al., 2011). According to the National Health and Nutrition Examination Survey in 2005, the median UIC in the United States was 164 μg/L and ranged from 154 to 173 μg/L, reflecting an iodine-sufficient state. The median UIC and prevalence of individuals with an excess of UIC >200 μg/L are higher in children between the age of 6–11 and subjects older than 70 years, as compared with other age groups (Caldwell et al., 2011). It has been reported that globally, 111 countries have data confirming the iodine-sufficient status, 30 countries are iodine-deficient, and 10 have excessive iodine intake (Pearce et al., 2013). Among the countries with a high iodine intake due to population-specific eating habits are Japan, Korea, and Canada (Pearce et al., 2013). Hence, these populations may particularly benefit from an LID. In fact, Ito et al. (2018), in a prospective study of 45 patients subjected to a strictly controlled low-iodine diet and a more liberalized self-controlled diet, revealed that the median urinary iodine–creatinine ratios at baseline were 286 μg/g Cr (range 40–7,100), while after the LID, they were significantly lower at 74 μg/g Cr (range 16–816). Interestingly, the National Health and Nutrition Examination Survey (NHANES) III study revealed higher all-cause mortality among individuals with excess iodine intake measured by UIE of more than 400 µg per day compared to individuals with adequate iodine intake (Inoue et al., 2018). It is to be noted that the European Association of Nuclear Medicine Therapy Committee recommended postponing therapy with RAI when the UIC exceeds 150–200 mcg/l (Luster et al., 2008). These recommendations could be met by adherence to the LID, particularly in populations with a high baseline iodine intake as even 4–5 days of a LID have shown to reduce UIE compared to nonrestricted diets (Maruca et al., 1984; Lakshmanan et al., 1988; Morris et al., 2001). Furthermore, UIEs were significantly less after 2 weeks of the LID compared to 1-week adherence to the LID, with mean values at 2 weeks being about 50% of those at 1 week (Park and Hennessey, 2004; Tomoda et al., 2005). This observation is particularly important for individuals with a high baseline UIE.

Nevertheless, the majority of the US population is characterized by UIE <200 µg/day, and therefore, a stringent LID might not be necessary. A less stringent LID may help reduce potential side effects of this diet. A large retrospective cohort study revealed a prevalence of hyponatremia in up to 2% of patients subjected to LID (Lee et al., 2014). The risk factors for hyponatremia are age >65 years, female gender, use of diuretics, and presence of multiple metastases that could contribute to SIADH (Li et al., 2016). Moreover, strict adherence to a LID may be associated with anxiety in some patients and can be time-consuming and difficult for medical teams to implement.

Another important factor that could determine the RAI ablation success rate is the method of TSH stimulation. However, a recent prospective cohort study including 94 patients from South Korea revealed that 1 week of a low-iodine diet is sufficient preparation for RAI, regardless of the method of TSH stimulation (Kang et al., 2019).

One of the strengths of our study is that we controlled for potential competing risks such as the age, tumor size, RAI dosage, and presence of distant metastases. Moreover, in this study, we evaluated a 24-h UIE, which is the gold standard for iodine status measurement (Baloch et al., 2003). Estimating UIE by the urinary iodine/creatinine ratio (µg/gCr) from spot urine samples is subject to error, particularly in patients with low muscle mass and protein intake and, consequently, low creatinine excretion.

Our study needs to be interpreted in the context of its limitations, which included a relatively small sample size and a median duration of a follow-up of 3.7 years. Confirmation of our findings in larger populations is needed. Although it would be worthwhile to analyze longer-term data, our study is the first to report the time to progression and is characterized by the longest follow-up duration among the published studies. Unfortunately, our study is underpowered to analyze overall survival due to the limited number of death events within the follow-up period (*n* = 1).

In conclusion, although an UIE exceeding 200 µg/day might be associated with a higher likelihood of progression in thyroid cancer patients treated with RAI, the presence of distant metastases at diagnosis is a stronger independent predictor of progression. A less stringent LID, such as avoiding seafood and seaweed, might be sufficient to achieve a UIE of <200 µg/day in the US population.

## Data Availability

The raw data supporting the conclusions of this article will be made available by the authors, without undue reservation.
