# Supplementary material for: The utility of low-iodine diet in preparation for thyroid cancer therapy with radioactive iodine—A cohort study
Source: Front Pharmacol. 2022 Sep 30;13:791710. doi: 10.3389/fphar.2022.791710 (PMC9562270; doi:10.3389/fphar.2022.791710)
Supplement: Supplementary file 1 [file Table1.DOCX]

| **Patient #** | **Location of persistent/recurrent disease** | **Evidence of progression** | | **Post-op Tg [ng/dl]** | **Suppressed Tg at the time of progression**  **[ng/dl]** | **Intervention** |
| --- | --- | --- | --- | --- | --- | --- |
|  |  | **At least a 20% increase in the sum of the diameters of target lesions** | **New lesions** |  |  |  |
| 1 | Pulmonary micro-and macro-metastases, bone metastases | No | Yes | 30.2 | >900 | RAI therapy 189 mCi (3^rd^ dose), cumulative dosage 524 mCi over 3 courses of RAI |
| 2 | Pulmonary micro and macro-metastases, central neck mass | No | Yes | N/A | 249.3 | Active surveillance |
| 3 | Peritoneal metastases from malignant struma ovarii | No | Yes | N/A | 99.3 | RAI therapy 148 mCi (2^nd^ dose), cumulative dosage 1015 mCi over 5 courses of RAI |
| 4 | Pulmonary micro-and macro-metastases, bone metastases | Yes | No | N/A | 59990 | RAI therapy with 400 mCi (2^nd^ dose), cumulative dosage 777 mCi over 3 courses of RAI |
| 5 | Pulmonary micro- and macro-metastases | No | Yes | 0.6 | 47.2 | RAI therapy 208 mCi (2^nd^ dose), cumulative dosage 359 mCi over 2 courses of RAI |
| 6 | Pulmonary micro and macro-metastases, central neck mass | No | Yes | >900 | >900 | Left central neck dissection |
| 7 | Lateral neck lymphadenopathy | No | Yes | N/A | 17.8 | RAI therapy 160 mCi (2^nd^ dose), cumulative dosage 589 mCi over 3 courses of RAI |
| 8 | Pulmonary micro-metastases and bone metastases | No | Yes | 54.1 | 434 | Active surveillance, zolendronic acid infusion |
| 9 | Pulmonary micro-and macro-metastases, bone metastases | No | Yes | >900 | >900 | RAI therapy 300 mCi (2^nd^ dose), cumulative dosage 400 mCi over 2 courses of RAI |
| 10 | Pulmonary micro-and macro-metastases, central neck mass | Yes | Yes | 11.6 | 17.2 | Right central neck dissection |
| 11 | Left lateral neck lymphadenopathy | Yes | No | N/A | 27.2 | Left lateral modified lymph node dissection |
| 12 | Left lateral neck lymphadenopathy | No | Yes | 8.4 | 12.1 | Left lateral modified lymph node dissection |
| 13 | Development of pulmonary micro- and macro-metastases | No | Yes | 21 | 3526 | RAI therapy 150 mCi (3rd dose), cumulative dosage 450 mCi over 3 courses of RAI |
| 14 | Central neck and mediastinal mass | Yes | No | <0.2 | 7.2 | RAI therapy 300 mCi (3rd dose), cumulative dosage 590 mCi over 3 courses of RAI |
| 15 | Pulmonary micro-and macro-metastases, bone metastases | Yes | No | 628 | 1374 | Active surveillance, zolendronic acid infusion |

**Supplemental Table 1. Characteristics of the patients presenting with disease progression.**
