# Supplementary figures and images for: The utility of low-iodine diet in preparation for thyroid cancer therapy with radioactive iodine—A cohort study
Source: Front Pharmacol. 2022 Sep 30;13:791710. doi: 10.3389/fphar.2022.791710 (PMC9562270; doi:10.3389/fphar.2022.791710)

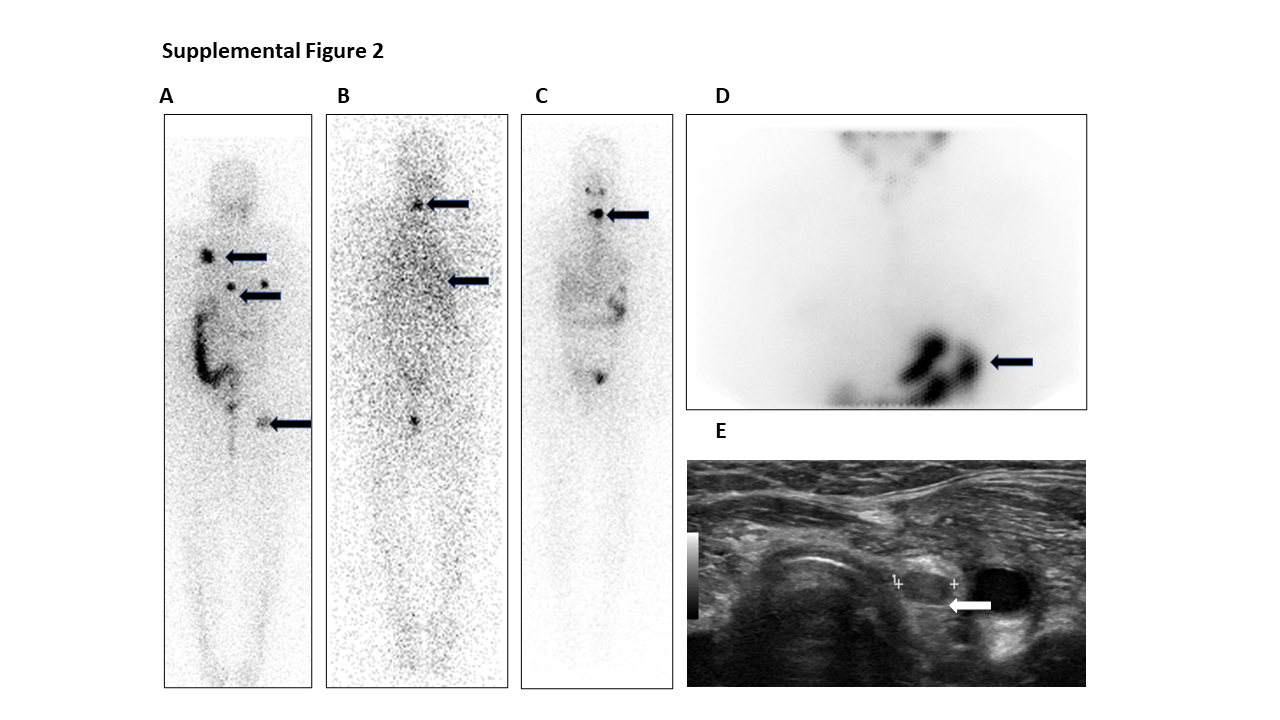

Supplement: Supplementary file 2 [file Image2.TIF]

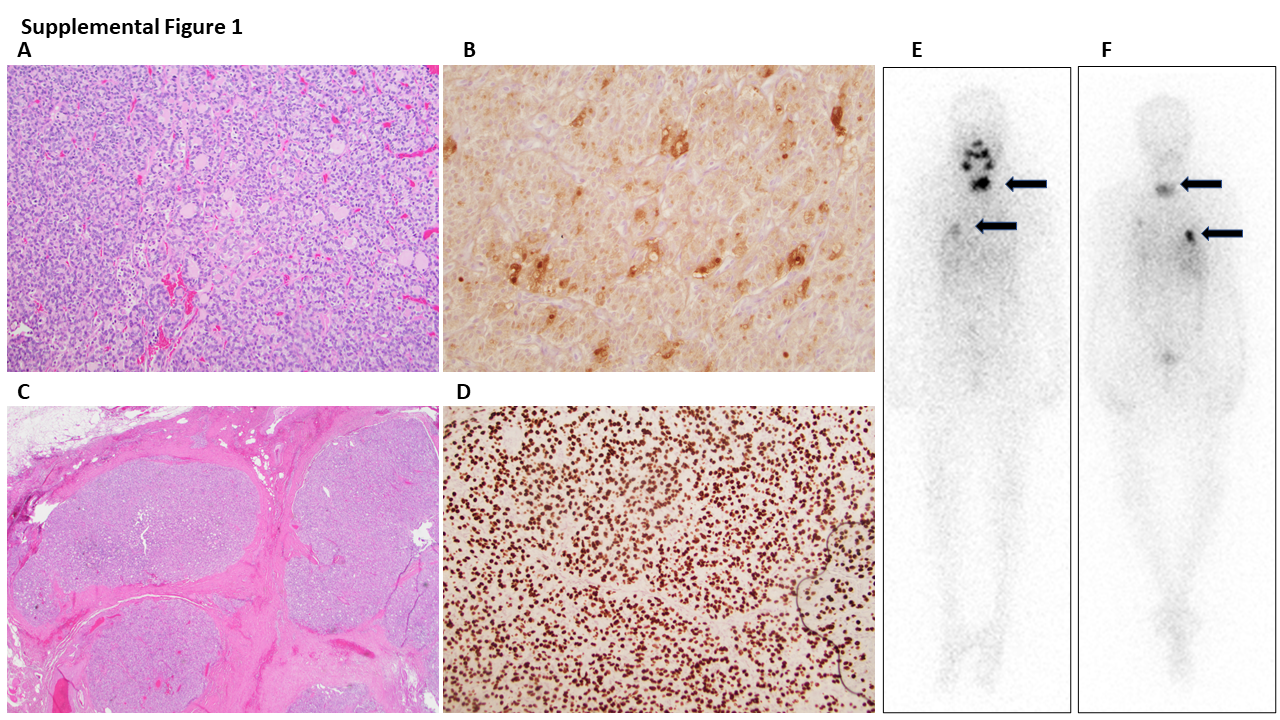

Supplement: Supplementary file 3 [file Image1.TIF]
